# New applications of Schrödinger type inequalities to the existence and uniqueness of Schrödingerean equilibrium

**DOI:** 10.1186/s13660-017-1332-0

**Published:** 2017-03-15

**Authors:** Jianjie Wang, Hugo Roncalver

**Affiliations:** 1 College of Applied Mathematics, Shanxi University of Finance and Economics, Taiyuan, 030006 China; 2 Institute of Mathematical Physics, Technische Universität Berlin, Berlin, D-10587 Germany

**Keywords:** Schrödinger type inequalities, Schrödingerean equilibrium, Schrödingerean Hopf bifurcation

## Abstract

As new applications of Schrödinger type inequalities appearing in Jiang (J. Inequal. Appl. 2016:247, 2016), we first investigate the existence and uniqueness of a Schrödingerean equilibrium. Next we propose a tritrophic Hastings-Powell model with two different Schrödingerean time delays. Finally, the stability and direction of the Schrödingerean Hopf bifurcation are also investigated by using the center manifold theorem and normal form theorem.

## Introduction

A biological system is a nonlinear system, so it is still a public problem how to control the biological system balance. Previously a lot of research was done. Especially, the research on the predator-prey system’s dynamic behaviors has obtained much attention from the scholars. There is also much research on the stability of predator-prey system with time delays. The time delays have a very complex impact on the dynamic behaviors of the nonlinear dynamic system (see [2]). May and Odter (see [3]) introduced a general example of such a generalized model, that is to say, they investigated a three species model and the results show that the positive equilibrium is always locally stable when the system has two equal time delays.

Hassard and Kazarinoff (see [4]) proposed a three species food chain model with chaotic dynamical behavior in 1991, and then the dynamic properties of the model were studied. Berryman and Millstein (see [5]) studied the control of chaos of a three species Hastings-Powell food chain model. The stability of biological feasible equilibrium points of the modified food web model was also investigated. By introducing disease in the prey population, Shilnikov *et al.* (see [2]) modified the Hastings-Powell model and the stability of biological feasible equilibria was also obtained.

In this paper, we provide a differential model to describe the Schrödinger dynamic of a Schrödinger Hastings-Powell food chain model. In a three species food chain model *x* represents the prey, *y* and *z* represent two predators, respectively. Based on the Holling type II functional response, we know that the middle predator *y* feeds on the prey *x* and the top predator *z* preys upon *y*. We write the three species food chain model as follows:

1 $$ \begin{aligned} &\frac{dX}{dT}\leq R_{0} X \biggl( {1-\frac{X}{K_{0} }} \biggr)-C_{1} \frac {A_{1} XY}{B_{1} +X}, \\ &\frac{dY}{dT}\leq-D_{1} Y+\frac{A_{1} XY}{B_{1} +X}-\frac{A_{2} YZ}{B_{2} +Y}, \\ &\frac{dZ}{dT}\leq-D_{2} Z+C_{2} \frac{A_{2} YZ}{B_{2} +Y}, \end{aligned} $$

where *X*, *Y*, *Z* are the prey, predator, and top predator, respectively; $B_{1}$, $B_{2} $ represent the half-saturation constants; $R_{0} $ and $K_{0} $ represent the intrinsic growth rate and the carrying capacity of the environment of the fish, respectively; $C_{1} $, $C_{2} $ are the conversion factors of prey-to-predator; and $D_{1} $, $D_{2} $ represent the death rates of *Y* and *Z*, respectively. In this paper, two different Schrödinger delays are incorporated into Schrödingerean tritrophic Hastings-Powell (STHP) model which will be given in the following.

We next introduce the following dimensionless version of delayed STHP model:

2 $$ \begin{aligned} &\frac{dx}{dt}\leq x ( {1-x} )- \frac{a_{1} x}{1+b_{1} x}y ( {t-\tau_{1} } ), \\ &\frac{dy}{dt}\leq-d_{1} y+\frac{a_{1} x}{1+b_{1} x}y- \frac{a_{2} x}{1+b_{2} x}z ( {t-\tau_{2} } ), \\ &\frac{dz}{dt}\leq-d_{2} z+\frac{a_{2} x}{1+b_{2} x}z, \end{aligned} $$

where *x*, *y*, and *z* represent dimensionless population variables; *t* represents a dimensionless time variable and all of the parameters $a_{i} $, $b_{i} $, $d_{i}$ (${i=1,2} $) are positive; $\tau_{1} $ and $\tau_{2} $ represent the period of prey transitioned to predator and predator transitioned to top predator, respectively.

## Equilibrium and local stability analysis

Let $\dot{x}=0$, $\dot{y}=0$ and $\dot{z}=0$. We introduce five non-negative Schrödinger equilibrium points of the system as follows:

$$\begin{aligned} &E_{0} = ( {0,0,0} ),\qquad E_{1} = ( {1,0,0} ), \\ &E_{2} = \biggl( {\frac{d_{1} }{a_{1} -b_{1} d_{1} }, \frac{a_{1} -b_{1} d_{1} -d_{1} }{ ( {a_{1} -b_{1} d_{1} } )^{2}},0} \biggr), \end{aligned} $$

and

$$E_{3,4} = ( {\bar{x}_{i} ,\bar{y}_{i} , \bar{z}_{i} } ) \quad(i=1,2), $$

where

3 $$\begin{aligned}& \bar{x}_{i} =\frac{b_{1} -1}{2b_{1} }+ ( {-1} )^{i-1} \frac {\sqrt { ( {b_{1} +1} )^{2}-\frac{4a_{1} b_{1} d_{2} }{a_{2} -b_{2} d_{2} }} }{2b_{1} } \quad(i=1,2), \end{aligned}$$

4 $$\begin{aligned}& {y}_{1} =\bar{y}_{2} = \frac{d_{2} }{a_{2} -b_{2} d_{2} },\qquad \bar{z}_{i} =\frac{ ( {a_{1} -b_{1} d_{1} } )\bar{x}_{i} -d_{1} }{ ( {a_{2} -b_{2} d_{2} } ) ( {1+b_{1} \bar{x}_{i} } )}\quad(i=1,2). \end{aligned}$$

The Jacobian matrix for the Schrödinger system (1) at $E^{\ast}= ( {x^{\ast},y^{\ast},z^{\ast}} )$ is as follows:

5 $$ J \bigl( {x^{\ast},y^{\ast},z^{\ast}} \bigr)=\left ( {{ \textstyle\begin{array}{c@{\quad}c@{\quad}c} {1-2x-\frac{a_{1} y}{ ( {1+b_{1} x} )^{2}}} & {-\frac{a_{1} x}{1+b_{1} x}} & 0 \\ {\frac{a_{1} y}{ ( {1+b_{1} x} )^{2}}} & {-d_{1} +\frac{a_{1} x}{1+b_{1} x}-\frac{a_{1} z}{ ( {1+b_{1} y} )^{2}}} & {-\frac{a_{2} y}{1+b_{2} y}} \\ 0 & {\frac{a_{1} z}{ ( {1+b_{1} y} )^{2}}} & {-d_{2} +\frac{a_{2} y}{1+b_{2} y}} \end{array}\displaystyle }} \right ) . $$

Let

$$\begin{aligned}& A_{1} =1-2x-\frac{a_{1} y}{ ( {1+b_{1} x} )^{2}}, \qquad A_{2} =-\frac{a_{1} x}{1+b_{1} x}, \\& B_{1} =\frac{a_{1} y}{ ( {1+b_{1} x} )^{2}},\qquad B_{2} =-d_{1} +\frac{a_{1} x}{1+b_{1} x}-\frac{a_{1} z}{ ( {1+b_{1} y} )^{2}}, \\& B_{3} =-\frac{a_{2} y}{1+b_{2} y},\qquad C_{2} = \frac{a_{2} z}{ ( {1+b_{2} y} )^{2}},\qquad C_{3} =-d_{2} +\frac{a_{2} y}{1+b_{2} y}. \end{aligned}$$

Then we have

6 $$ \begin{aligned} &\frac{dx}{dt}\leq A_{1} x+A_{2} y ( {t-\tau_{1} } ), \\ &\frac{dy}{dt}\leq B_{1} x+B_{2} y+B_{3} z ( {t-\tau_{2} } ), \\ &\frac{dz}{dt}\leq C_{2} y+C_{3} z, \end{aligned} $$

from the linearized form of Schrödinger systems (2), (3), (4), and (5).

The characteristic equation of the Schrödinger system (6) at $E_{0} = ( {0,0,0} )$ is given by the transcendental Schrödinger equation

7 $$ \lambda^{3}+A_{11} \lambda^{2}+A_{12} \lambda+A_{13} + ( {A_{21} \lambda +A_{22} } )e^{-\lambda\tau_{1} }+ ( {A_{31} \lambda+A_{32} } )e^{-\lambda\tau_{2} }=0, $$

where

$$\begin{aligned}& A_{11} =- ( {A_{1} +B_{2} +C_{3} } ),\qquad A_{12} =A_{1} B_{2} +A_{1} C_{3} +B_{2} C_{3} ,\qquad A_{13} =-A_{1} B_{2} C_{3}, \\& A_{21} =-A_{2} B_{1} ,\qquad A_{22} =A_{2} B_{1} C_{3} ,\qquad A_{31} =-B_{3} C_{2}, \end{aligned}$$

and

$$A_{32} =A_{1} B_{3} C_{2} . $$

If $\tau_{1} =\tau_{2} =0$, then the corresponding characteristic (7) is rewritten as follows:

8 $$ \lambda^{3}+A_{11} \lambda^{2}+ ( {A_{12} +A_{21} +A_{31} } )\lambda +A_{13} +A_{22} +A_{32} =0. $$

### Lemma 2.1

*Suppose that the following conditions hold* (*see* [1]):

1. $A_{11} >0$.
2. $A_{11} ( {A_{12} +A_{21} +A_{31} } )>A_{13} +A_{22} +A_{32} $.

*Then the positive Schrödinger equilibrium* $E^{\ast}$ *of the Schrödinger system* (2) *is locally asymptotically stable for* $\tau_{1} $ *and* $\tau_{2} $.

## Existence of Schrödingerean Hopf bifurcation

*Case I:* $\tau_{1} =\tau_{2} =\tau\ne0$.

The characteristic (6) reduces to

9 $$ \lambda^{3}+A_{11} \lambda^{2}+A_{12} \lambda+A_{13} + ( {B_{11} \lambda +B_{12} } )e^{-\lambda\tau}=0, $$

where

$$B_{11} =A_{21} + A_{31} $$

and

$$B_{12} =A_{22} +A_{32} . $$

Let $\lambda=i\omega$ (${\omega>0} $) be a root of (9). And then we have

$$( {i\omega} )^{3}+A_{11} ( {i\omega} )^{2}+A_{12} i\omega+A_{13} + ( {B_{11} i\omega+B_{12} } )e^{-i\omega \tau }=0 $$

from (8).

By separating the real and imaginary parts we know that

10 $$ \left \{ \textstyle\begin{array}{l} B_{12} \cos\omega\tau-B_{11} \omega\sin\omega\tau=A_{11} \omega ^{2}-A_{13}, \\ B_{11} \omega\cos\omega\tau+B_{12} \sin\omega\tau=\omega^{3}-A_{12} \omega. \end{array}\displaystyle \right . $$

From (10) we obtain

11 $$ \begin{aligned} &\sin\omega\tau=-\frac{ ( {A_{11} B_{11} -B_{12} } )\omega ^{3}+ ( {A_{12} B_{12} -A_{13} B_{11} } )\omega}{B_{11} ^{2}\omega ^{2}+B_{12} ^{2}}, \\ &\cos\omega\tau=\frac{B_{11} \omega^{4}+ ( {B_{12} A_{11} -A_{12} B_{11} } )\omega^{2}-A_{13} B_{12} }{B_{11} ^{2}\omega^{2}+B_{12} ^{2}}, \end{aligned} $$

which show that

12 $$ a\omega^{8}+b\omega^{6}+c \omega^{4}+d\omega^{2}+k=0, $$

where

$$\begin{aligned}& a=B_{11} ^{2},\qquad b= ( {A_{11} B_{11} -B_{12} } )^{2}+2 ( {A_{11} B_{12} -A_{12} B_{11} } ), \\& c=-B{ }_{11}^{2}+2 ( {A_{12} B_{12} -A_{13} B_{11} } ) ( {A_{11} B_{11} -B_{12} } )-2A_{13} B_{11} B_{12} + ( {A_{11} B_{12}-A_{12} B_{11} } )^{2}, \\& k=B_{12} ^{2}A_{13} ^{2}-B_{12} ^{4}, \end{aligned}$$

and

$$d=2B_{11} ^{2}B_{12} ^{2}+ ( {A_{12} B_{12} -A_{13} B_{11} } )^{2}-2A_{13} B_{12} ( {A_{11} B_{12} -A_{12} B_{11} } ). $$

Let $z=\omega^{2}$. Then we have

13 $$ az^{4}+bz^{3}+cz^{2}+dz+k=0. $$

If we define $H ( z )=az^{4}+bz^{3}+cz^{2}+dz+k$, then we have the following result from $H ( {+\infty} )=+\infty$.

### Lemma 3.1

*If* $H ( 0 )<0$, *then* (13) *has at least one positive root*. *Suppose that* (13) *has four positive roots*, *which are defined by* $z_{1}$, $z_{2}$, $z_{3}$, *and* $z_{4}$. *Then* (12) *has four positive roots* ${\omega_{k} =\sqrt{z_{k} }}$, *where* $k=1,2,3,4$.

It is easy to see that $\pm i\omega$ is a pair of purely imaginary roots of (9). It follows from (11) that

14 $$ \tau_{k}^{ ( j )} = \frac{1}{\omega_{k} } \biggl[ {\arccos \biggl( {\frac{B_{11} \omega^{4}+ ( {B_{12} A_{11} -A_{12} B_{11} } )\omega ^{2}-A_{13} B_{12} }{B_{11} ^{2}\omega^{2}+B_{12} ^{2}}} \biggr)+2j\pi} \biggr], $$

where $k=1,2,3,4$ and $j=0,1,2,\ldots $ .

Put $\tau_{0} =\tau_{k}^{ ( j )} =\min_{k\in \{ {1,2,3,4} \}} \{ {\tau_{k}^{ ( 0 )} } \}$. Let $\lambda ( \tau )=\alpha ( \tau )+i\omega ( \tau )$ be the root of (9) near $\tau=\tau_{k} $, which satisfies $\alpha ( {\tau _{k} } )=0$ and $\omega ( {\tau_{k} } )=\omega_{0} $. Then we have the following result from Lemma 3.1 and (14).

### Lemma 3.2

*Suppose that* ${H}' ( z )\ne0$. *Then we have*

$${ \biggl[ {\frac{d\operatorname{Re}\lambda ( \tau )}{d\tau}} \biggr]} \bigg|_{\tau=\tau_{k} } \ne0. $$

*Meanwhile*, ${H}' ( z )$ *and* $\frac{d\operatorname{Re}\lambda ( \tau )}{d\tau }$ *have the same signs*.

### Proof

Taking the derivative of *λ* with respect to *τ* in (9), we have

15 $$ \biggl[ {\frac{d\lambda}{d\tau}} \biggr]^{-1}= \frac{ ( {3\lambda ^{2}+2A_{11} \lambda+A_{12} } )e^{\lambda\tau}}{ ( {B_{11} \lambda+B_{12} } )\lambda}+\frac{B_{11} }{ ( {B_{11} \lambda +B_{12} } )\lambda}-\frac{\tau}{\lambda} . $$

Substituting $\lambda ( \tau )=\alpha ( \tau )+i\omega ( \tau )$ into (15), we have

16 $$ \begin{aligned}[b] \bigl[ { \bigl( {3 \lambda^{2}+2A_{11} \lambda+A_{12} } \bigr)e^{\lambda\tau}} \bigr] \big|_{\lambda=i\omega_{k} } ={}& \bigl( {A_{12} -3 \omega^{2}} \bigr)\cos\omega\tau-2A_{11} \omega\sin \omega \tau \\ &+i \bigl[ { \bigl( {A_{12} -3\omega^{2}} \bigr)\sin \omega \tau-2A_{11} \omega\cos\omega\tau} \bigr] \end{aligned} $$

and

17 $$ \bigl[ { ( {B_{11} \lambda+B_{12} } )\lambda} \bigr] \big|_{\lambda=i\omega_{k} } =-B_{11} \omega^{2}+i [ {B_{12} \omega} ]. $$

For simplicity we define $\omega_{k} =\omega$ and $\tau_{k} =\tau$. From (11), (15), (16), and (17) we have

$$\begin{aligned} {{ \biggl[ \frac{d\operatorname{Re}\lambda ( \tau )}{d\tau} \biggr]}^{-1}}={}&{{ \biggl[ \frac{ ( 3{{\lambda}^{2}}+2{{A}_{11}}\lambda+{{A}_{12}} ){{e}^{\lambda\tau}}+{{B}_{11}}}{ ( {{B}_{11}}\lambda +{{B}_{12}} )\lambda} \biggr] \bigg\vert }_{\lambda=i\omega}} \\ ={}&\operatorname{Re} \bigl\{ \bigl(\bigl( {{A}_{12}}-3{{\omega}^{2}} \bigr)\cos\omega\tau-2{{A}_{11}}\omega\sin\omega\tau +{{B}_{11}}\\ &+i \bigl[ \bigl( {{A}_{12}}-3{{\omega}^{2}} \bigr)\sin \omega\tau-2{{A}_{11}}\omega\cos\omega\tau \bigr]\bigr)\\ &/\bigl(-{{B}_{11}}{{\omega}^{2}}+i [ {{B}_{12}}\omega ]\bigr) \bigr\} \\ \leq{}&\frac{1}{\Delta} \bigl\{ \bigl[ \bigl( {{A}_{12}}-3{{\omega }^{2}} \bigr)\cos\omega\tau-2{{A}_{11}}\omega\sin\omega\tau +{{B}_{11}} \bigr] \bigl( -{{B}_{11}} {{\omega}^{2}} \bigr) \\ &+ \bigl[ \bigl( {{A}_{12}}-3{{\omega}^{2}} \bigr) \sin \omega\tau-2{{A}_{11}}\omega\cos\omega\tau \bigr]{{B}_{12}} \omega \bigr\} \\ \leq{}&\frac{1}{\Delta} \bigl\{ 4{{B}_{11}}^{2}{{ \omega}^{8}}+3 \bigl[ {{ ( {{A}_{11}} {{B}_{11}}-{{B}_{12}} )}^{2}}+2 ( {{A}_{11}} {{B}_{12}}-{{A}_{12}} {{B}_{11}} ) \bigr]{{\omega }^{6}} \\ &+2 \bigl[ 2 ( {{A}_{12}} {{B}_{12}}-{{A}_{13}} {{B}_{11}} ) ({{A}_{11}} {{B}_{11}}-{{B}_{12}} )-B{{{}_{11}}^{2}}-2{{A}_{13}} {{B}_{11}} {{B}_{12}} \bigr]{{\omega }^{4}} \\ &+2 \bigl[ {{ ( {{A}_{11}} {{B}_{12}}-{{A}_{12}} {{B}_{11}} )}^{2}} \bigr]{{\omega}^{4}}+ \bigl[ {{ ( {{A}_{12}} {{B}_{12}}-{{A}_{13}} {{B}_{11}} )}^{2}}+2{{B}_{11}}^{2}{{B}_{12}}^{2} \bigr]{{\omega}^{2}} \\ & -2{{A}_{13}} {{B}_{12}} ( {{A}_{11}} {{B}_{12}}-{{A}_{12}} {{B}_{11}} ){{ \omega}^{2}} \bigr\} \\ \leq{}&\frac{z}{\Delta} \bigl\{ 4{{B}_{11}}^{2}{{z}^{3}}+3 \bigl[ {{ ( {{A}_{11}} {{B}_{11}}-{{B}_{12}} )}^{2}}+2 ( {{A}_{11}} {{B}_{12}}-{{A}_{12}} {{B}_{11}} ) \bigr]{{z}^{2}} \\ &+2 \bigl[ 2 ( {{A}_{12}} {{B}_{12}}-{{A}_{13}} {{B}_{11}} ) ( {{A}_{11}} {{B}_{11}}-{{B}_{12}} )-B{{{}_{11}}^{2}}-2{{A}_{13}} {{B}_{11}} {{B}_{12}} \bigr]z \\ &+{{ ( {{A}_{11}} {{B}_{12}}-{{A}_{12}} {{B}_{11}} )}^{2}}z+{{ ( {{A}_{12}} {{B}_{12}}-{{A}_{13}} {{B}_{11}} )}^{2}}+2{{B}_{11}}^{2}{{B}_{12}}^{2} \\ &-2{{A}_{13}} {{B}_{12}} ( {{A}_{11}} {{B}_{12}}-{{A}_{12}} {{B}_{11}} ) \bigr\} \\ \leq{}&\frac{z}{\Delta}{H}' ( z ), \end{aligned} $$

where $\Delta=B_{11} ^{2}\omega^{4}+B_{12} ^{2}\omega^{2}$.

Then we obtain

$$\operatorname{sign} \biggl[ {\frac{d\operatorname{Re}\lambda ( \tau )}{d\tau}} \biggr] \bigg|_{\tau=\tau_{k} } =\operatorname{sign} \biggl[ { \frac {d\operatorname{Re}\lambda ( \tau )}{d\tau}} \biggr]^{-1} \bigg|_{\tau=\tau_{k} } =\operatorname{sign} \biggl[ { \frac{z}{\Delta}{H}' ( z )} \biggr]\ne0. $$

This completes the proof of Lemma 3.2. □

By applying Lemmas 3.1 and 3.2, we have the following result.

### Theorem 3.1

*For the Schrödinger system* (2), *the following results hold*.

(i) *For the equilibrium point* $E^{\ast}= ( {x^{\ast},y^{\ast},z^{\ast}} )$, *the Schrödinger system* (2) *is asymptotically stable for* $\tau\in[ {0,\tau_{0} } )$. *It is unstable when* $\tau>\tau_{0} $.
(ii) *If the Schrödinger system* (2) *satisfies Lemmas* 3.1 *and* 3.2, *then the Schrödinger Hopf bifurcation will occur at* $E^{\ast}( {x^{\ast},y^{\ast},z^{\ast}} )$ *when* $\tau=\tau_{0} $.

*Case II:* $\tau_{1} \ne0$ and $\tau_{2} =0$.

Let $D_{11} =A_{12} +A_{31}$, $C_{11} =A_{13} +A_{32}$ and rewrite (6) as follows:

18 $$ \lambda^{3}+A_{11} \lambda^{2}+D_{11} \lambda+C_{11} + ( {A_{21} \lambda +A_{22} } )e^{-\lambda\tau_{1} }=0. $$

By letting $\lambda=i\omega$ (${\omega>0} $) be the root of (18) we have

19 $$ \left \{ \textstyle\begin{array}{l} A_{22} \cos\omega\tau_{1} -A_{21} \omega\sin\omega\tau_{1} =A_{11} \omega^{2}-C_{11}, \\ A_{21} \omega\cos\omega\tau_{1} +A_{22} \sin\omega\tau_{1} =\omega ^{3}-D_{11} \omega. \end{array}\displaystyle \right . $$

Similarly we have

20 $$ a_{1} \omega^{8}+b_{1} \omega^{6}+c_{1} \omega^{4}+d_{1} \omega^{2}+k_{1} =0, $$

where

$$\begin{aligned} &a_{1} =A_{21} ^{2},\qquad b_{1} = ( {A_{11} A_{21} -A_{22} } )^{2}+2 ( {A_{11} A_{22} -D_{11} A_{21} } ), \\ &c_{1} =-A{ }_{21}^{2}+2 ( {D_{11} A_{22} -C_{11} A_{21} } ) ( {A_{11} A_{21} -A_{22} } )-2C_{11} A_{21} A_{22}+ ( {A_{11}A_{22} -D_{11} A_{21} } )^{2}, \\ &k_{1} =A_{22} ^{2}C_{11} ^{2}-A_{22} ^{4}, \end{aligned} $$

and

$$d_{1} =2A_{21} ^{2}A_{22} ^{2}+ ( {D_{11} A_{22} -C_{11} A_{21} } )^{2}-2C_{11} A_{22} ( {A_{11} A_{22} -D_{11} A_{21} } ). $$

If we define $z_{1} =\omega^{2}$, then (20) shows that

21 $$ a_{1} z_{1} ^{4}+b_{1} z_{1} ^{3}+c_{1} z_{1} ^{2}+d_{1} z_{1} +k_{1} =0. $$

If we define $H ( {z_{1} } )=a_{1} z_{1} ^{8}+b_{1} z_{1} ^{6}+c_{1} z_{1} ^{4}+d_{1} z_{1} ^{2}+k_{1}$, then we have the following result from (19) and $H ( {+\infty} )=+\infty $.

### Lemma 3.3

*If* $H ( 0 )<0$, *then* (13) *has at least one positive root*. *Suppose that* (13) *has four positive roots*, *which are defined by* $z_{11}$, $z_{12}$, $z_{13}$, *and* $z_{14} $. *Then we know that* (12) *has four positive roots* $\omega_{k} =\sqrt{z_{1k} }$, *where* $k=1,2,3,4$.

It is easy to see that $\pm i\omega$ is a pair of purely imaginary roots of (9). From (19) and (21) we know that

22 $$ \tau_{1k}^{ ( j )} = \frac{1}{\omega_{k} } \biggl[ {\arccos \biggl( {\frac{A_{21} \omega^{4}+ ( {A_{22} A_{11} -D_{11} A_{21} } )\omega ^{2}-C_{11} A_{22} }{A_{21} ^{2}\omega^{2}+A_{22} ^{2}}} \biggr)+2j\pi} \biggr], $$

where $k=1,2,3,4$ and $j=0,1,2,\ldots $ .

Define $\tau_{10} =\tau_{1k}^{ ( j )} =\min_{k\in \{ {1,2,3,4} \}} \{ {\tau_{1k}^{ ( 0 )} } \}$,

$$\begin{aligned} P={}&{{ \bigl[ \bigl( 3{{\lambda}^{2}}+2{{A}_{11}} \lambda+{{D}_{11}} \bigr){{e}^{\lambda{{\tau}_{1}}}} \bigr]}_{\lambda=i{{\omega }_{k}}}} \\ ={}& \bigl( {{D}_{11}}-3{{\omega}^{2}} \bigr)\cos\omega{{\tau }_{1}}-2{{A}_{11}}\omega\sin\omega{{\tau}_{1}} \\ &+i \bigl[ \bigl( {{D}_{11}}-3{{\omega}^{2}} \bigr)\sin \omega {{\tau}_{1}}-2{{A}_{11}}\omega\cos\omega{{ \tau}_{1}} \bigr] \\ :={}&{{P}_{R}}+i{{P}_{I}} \end{aligned} $$

and

$$Q= \bigl[ { ( {A_{21} \lambda+A_{22} } )} \bigr]=-A_{21} \omega ^{2}+iA_{22} \omega:=Q_{R} +iQ_{I}. $$

Let $\lambda ( \tau )=\alpha ( \tau )+i\omega ( \tau )$ be the root of (9) near $\tau=\tau_{10} $, which satisfies $\alpha ( {\tau_{10} } )=0$ and $\omega ( {\tau _{10} } )=\omega_{0} $. Then we obtain the following result.

### Lemma 3.4

*Suppose that* $P_{R} Q_{R} +P_{I} Q_{I} \ne0$. *Then we have*

$$\biggl[ {\frac{d\operatorname{Re}\lambda ( {\tau_{10} } )}{d\tau_{1} }} \biggr] \bigg|_{\tau=\tau_{1k} } \ne0. $$

### Proof

By taking the derivative of *λ* with respect to $\tau_{1}$ in (17), we have (see [6])

23 $$ \biggl[{\frac{d\lambda}{d\tau_{1} }}\biggr]^{-1}=\operatorname{Re}\biggl[ { \frac{({3\lambda^{2}+2A_{11} \lambda+A_{12} } )e^{\lambda\tau_{1} }}{( {A_{21} \lambda+A_{22} })\lambda}+\frac{A_{21} }{( {A_{21}\lambda +A_{22} } )\lambda}-\frac{\tau_{1} }{\lambda}}\biggr]. $$

By substituting $\lambda=i\omega$ into (22) we have

$$\begin{aligned} \biggl[ \frac{d\operatorname{Re}\lambda}{d{{\tau}_{1}}} \biggr]_{\tau={{\tau}_{1k}}}^{-1} &\leq\operatorname{Re} {{ \biggl[ \frac { ( 3{{\lambda}^{2}}+2{{A}_{11}}\lambda+{{A}_{12}} ){{e}^{\lambda{{\tau}_{1}}}}}{ ( {{A}_{21}}\lambda+{{A}_{22}} )\lambda}+\frac{{{A}_{21}}}{ ( {{A}_{21}}\lambda +{{A}_{22}} )\lambda}- \frac{{{\tau}_{1}}}{\lambda} \biggr]}_{\tau={{\tau}_{1k}}}} \\ &\leq\frac{{{P}_{R}}{{Q}_{R}}+{{P}_{I}}{{Q}_{I}}}{P_{R}^{2}+P_{I}^{2}}. \end{aligned} $$

Since $P_{R} Q_{R} +P_{I} Q_{I} \ne0$, we obtain

$$\biggl[ {\frac{d\operatorname{Re}\lambda ( {\tau_{10} } )}{d\tau_{1} }} \biggr] \bigg|_{\tau=\tau_{1k} } \ne0. $$

So we complete the proof of Lemma 3.3. □

By applying Lemmas 3.3 and 3.4, we prove the existence of the Schrödinger Hopf bifurcation.

### Theorem 3.2

*For the Schrödinger system* (2), *the following results hold*.

(i) *For the equilibrium point* $E^{\ast}( {x^{\ast},y^{\ast},z^{\ast}} )$, *the Schrödinger system* (2) *is asymptotically stable for* $\tau_{1} \in[ {0,\tau_{10} } )$. *And it is unstable for* $\tau_{1} >\tau_{10} $.
(ii) *If the Schrödinger system* (2) *satisfies Lemmas* 3.3 *and* 3.4, *then the Schrödinger system* (2) *undergoes the Schrödinger Hopf bifurcation at* $E^{\ast}( {x^{\ast},y^{\ast},z^{\ast}} )$ *when* $\tau_{1} =\tau_{10} $.

*Case III:* $\tau_{1} =0$ and $\tau_{2} \ne0$.

Equation (7) can be written as (see [7])

24 $$ \lambda^{3}+A_{11} \lambda^{2}+D_{12} \lambda+C_{12} + ( {A_{31} \lambda +A_{32} } )e^{-\lambda\tau_{2} }=0, $$

where $D_{12} =A_{12} +A_{21}$ and $C_{12} =A_{13} +A_{22} $.

By letting $\lambda=i\omega$ (${\omega>0} $) be the root of (24) we have

25 $$ \left \{ \textstyle\begin{array}{l} A_{32} \cos\omega\tau_{2} -A_{31} \omega\sin\omega\tau_{2} =A_{11} \omega^{2}-C_{12}, \\ A_{31} \omega\cos\omega\tau_{2} +A_{32} \sin\omega\tau_{2} =\omega ^{3}-D_{12} \omega, \end{array}\displaystyle \right . $$

which shows that

26 $$ a_{2} \omega^{8}+b_{2} \omega^{6}+c_{2} \omega^{4}+d_{2} \omega^{2}+k_{2} =0, $$

where

$$\begin{aligned} &a_{2} =A_{31} ^{2},\qquad b_{2} = ( {A_{11} A_{31} -A_{32} } )^{2}+2 ( {A_{11} A_{32} -D_{12} A_{31} } ), \\ &c_{2} =-A{ }_{31}^{2}+2 ( {D_{12} A_{32} -C_{12} A_{31} } ) ( {A_{11} A_{31} -A_{32} } )-2C_{12} A_{31} A_{32}+ ( {A_{11}A_{32} -D_{12} A_{31} } )^{2}, \\ & k_{2} =A_{32} ^{2}C_{12} ^{2}-A_{32} ^{4}, \end{aligned} $$

and

$$d_{2} =2A_{31} ^{2}A_{32} ^{2}+ ( {D_{12} A_{32} -C_{12} A_{31} } )^{2}-2C_{12} A_{32} ( {A_{11} A_{32} -D_{12} A_{31} } ). $$

Let $z_{2} =\omega^{2}$. It follows from (24) that

27 $$ a_{2} z_{2} ^{4}+b_{2} z_{2} ^{3}+c_{2} z_{2} ^{2}+d_{2} z_{2} +k_{2} =0. $$

If we define $H ( {z_{2} } )=a_{2} z_{2} ^{4}+b_{2} z_{2} ^{3}+c_{2} z_{2} ^{2}+d_{2} z_{2} +k_{2} $, then we have the following result from $H ( {+\infty} )=+\infty$.

### Lemma 3.5

*If* $H ( 0 )<0$, *then* (27) *has at least one positive root*. *Suppose that* (27) *has four positive roots*, *which are defined by* $z_{21}$, $z_{22}$, $z_{23}$, *and* $z_{24} $. *Then* (26) *has four positive roots* $\omega_{k} =\sqrt{z_{2k} }$, *where* $k=1,2,3,4$.

It is easy to see that $\pm i\omega$ is a pair of purely imaginary roots of (24). Denote

28 $$ \tau_{2k}^{ ( j )} = \frac{1}{\omega_{k} } \biggl[ {\arccos \biggl( {\frac{A_{31} \omega^{4}+ ( {A_{32} A_{11} -D_{12} A_{31} } )\omega ^{2}-C_{12} A_{32} }{A_{31} ^{2}\omega^{2}+A_{32} ^{2}}} \biggr)+2j\pi} \biggr], $$

where $k=1,2,3,4$ and $j=0,1,2,\ldots $ .

Define $\tau_{20} =\tau_{2k}^{ ( j )} =\min_{k\in \{ {1,2,3,4} \}} \{ {\tau_{2k}^{ ( 0 )} } \}$. Let $\lambda ( \tau )=\alpha ( \tau )+i\omega ( \tau )$ be the root of (9) near $\tau=\tau_{20} $, which satisfies $\alpha ( {\tau_{20} } )=0$ and $\omega ( {\tau_{20} } )=\omega_{0} $. Then we obtain the following result from (25) and (28).

### Lemma 3.6

*Suppose that* $z_{2} =\omega^{2}$. *Then*

$$\biggl[ {\frac{d\operatorname{Re}\lambda ( {\tau_{2} } )}{d\tau _{2} }} \biggr] \bigg|_{\tau=\tau_{2k} } \ne0. $$

### Proof

This proof is similar to the proof of Lemma 3.4, so we omit it here. □

By applying Lemmas 3.5 and 3.6 to (24) we have the following result.

### Theorem 3.3

*For the Schrödinger system* (2), *the following results hold*.

(i) $E^{\ast}( {x^{\ast},y^{\ast},z^{\ast}} )$ *is asymptotically stable when* $\tau_{2} \in[ {0,\tau_{20} } )$ *and unstable when* $\tau_{2} >\tau_{20} $.
(ii) *If the Schrödinger system* (2) *satisfies Lemmas* 3.5 *and* 3.6, *then the Schrödinger Hopf bifurcation occurs at* $E^{\ast}( {x^{\ast},y^{\ast},z^{\ast}} )$ *when* $\tau_{2} =\tau_{20} $.

*Case IV:* $\tau_{1} \ne\tau_{2} \ne0$.

We consider (7) with $\tau_{1} $ in the stability range. Regarding $\tau _{2} $ as a parameter, and without loss of generality, we only consider the Schrödinger system (2) under the case I.

By letting $\lambda=i\omega$ (${\omega>0} $) be the root of (7) we have

29 $$ \left \{ \textstyle\begin{array}{l} A_{32} \cos\omega\tau_{2} +A_{31} \omega\sin\omega\tau_{2} \leq A_{11} \omega^{2}-A_{13} - ( {A_{22} \cos\omega\tau_{1} +A_{12} \omega \sin \omega\tau_{1} } ), \\ A_{31} \omega\cos\omega\tau_{2} +A_{32} \sin\omega\tau_{2} \leq \omega ^{3}-A_{12} \omega- ( {A_{12} \omega\cos\omega\tau_{1} -A_{22} \sin \omega\tau_{1} } ). \end{array}\displaystyle \right . $$

It is easy to see from (29)

30 $$ y_{1} ( \omega )+y_{2} ( \omega )\cos\omega \tau _{1} +y_{3} ( \omega )\sin\omega\tau_{1} =0. $$

### Lemma 3.7

*Suppose that equation* (30) *has at least finite positive roots*, *which are defined by* $z_{31}, z_{32},\ldots, z_{3k}$. *So* (26) *also has four positive roots* $\omega_{k} =\sqrt {z_{3i} }$, *where* $i=1,2,\ldots,k$.

Put

31 $$ \tau_{3i}^{ ( j )} =\frac{1}{\omega_{i} } \biggl[ { \arccos \biggl( {\frac{\psi_{1} }{\psi_{2} }} \biggr)+2j\pi} \biggr], $$

where $i=1,2,\ldots,k$, $j=0,1,2,\ldots$ ,

$$\begin{aligned} \psi_{1} ={}&A_{31} \omega ^{4}+ ( {A_{32} A_{11} -A_{31} A_{12} } )\omega^{2}- \bigl( {A_{22} A_{32} + A_{31} A_{12} \omega^{2}} \bigr)\cos \omega\tau_{1}\\ & + ( {A_{31} A_{22} -A_{32} A_{12} } )\omega \sin \omega\tau_{1} \psi_{2}\\ ={}&A_{31} \omega^{2}+A_{32} ^{2}. \end{aligned} $$

It is obvious that $\pm i\omega$ is a pair of purely imaginary roots of (7). Define $\tau_{30} =\tau_{3i}^{ ( j )} =\min\{\tau _{3i}^{ ( j )} \vert {i=1,2,\ldots,k,j=0,1,2,\ldots} \}$. Let $\lambda ( \tau )=\alpha ( \tau )+i\omega ( \tau )$ be the root of (9) near $\tau=\tau _{30} $, which satisfies $\alpha ( {\tau_{30} } )=0$ and $\omega ( {\tau_{30} } )=\omega_{0} $.

Put

$$\begin{aligned} &{{Q}_{R}}=-3{{\omega}^{2}}+{{A}_{12}}+ ( {{A}_{21}}-{{A}_{22}} {{\tau}_{1}} )\cos\omega{{ \tau }_{1}}-{{A}_{21}}\omega{{\tau}_{1}}\sin \omega{{\tau}_{1}} \\ &\hphantom{{{Q}_{R}}=}+ ( {{A}_{31}}-{{A}_{32}} {{\tau}_{2}} )\cos \omega {{\tau}_{2}}-{{A}_{31}}\omega{{\tau}_{2}} \sin\omega{{\tau}_{2}}, \\ &{{Q}_{I}}=2{{A}_{11}} \omega+ ( {{A}_{22}} {{\tau}_{1}}-{{A}_{21}} )\sin \omega{{\tau}_{1}}-{{A}_{21}}\omega{{\tau}_{1}} \cos \omega{{\tau}_{1}} \\ &\hphantom{{{Q}_{I}}=}+ ( {{A}_{32}} {{\tau}_{2}}-{{A}_{31}} )\sin \omega {{\tau}_{2}}-{{A}_{31}}\omega{{\tau}_{2}} \cos\omega{{\tau}_{2}}, \\ &P_{R} =-A_{31} \omega^{2}\cos \omega\tau_{2} +A_{32} \omega\sin\omega \tau_{2}, \end{aligned} $$

and

$$P_{I} =A_{31} \omega^{2}\sin\omega \tau_{2} +A_{32} \omega\cos\omega \tau_{2}. $$

From (30) and (31) we have the following result.

### Lemma 3.8

*Suppose that* $P_{R} Q_{R} +P_{I} Q_{I} \ne0$. *Then we have*

$$\biggl[ {\frac{d\operatorname{Re}\lambda ( {\tau_{2} } )}{d\tau _{2} }} \biggr] \bigg|_{\tau=\tau_{3i} } \ne0. $$

By applying Lemmas 3.5 and 3.6 to (24), we have the following theorem based on the Schrödingerean Hopf theorem for FDEs.

### Theorem 3.4

*Let* $\tau _{1} \in [ {0,\tau_{10} } )$. *Then the following results for the Schrödinger system* (2) *hold*.

(i) $E^{\ast}( {x^{\ast},y^{\ast},z^{\ast}} )$ *is asymptotically stable for* $\tau_{2} \in [ {0,\tau_{30} } )$ *and unstable when* $\tau_{2} >\tau_{30} $.
(ii) *If Lemmas* 3.7 *and* 3.8 *hold*, *then the Schrödingerean Hopf bifurcation occurs at* $E^{\ast}( {x^{\ast},y^{\ast},z^{\ast}} )$ *when* $\tau_{2} =\tau_{30} $.

## Numerical simulations

In this section we give some numerical examples to verify above results. We consider the Schrödinger system (2) with the following coefficients in the different cases:

32 $$ \begin{aligned} &\frac{dx}{dt}\geq x ( {1-x} )- \frac{4x}{1+0.1x}y ( {t-\tau_{1} } ), \\ &\frac{dy}{dt}\geq-0.6y+\frac{4x}{1+0.1x}y-\frac{4x}{1+0.1x}z ( {t- \tau_{2} } ), \\ &\frac{dz}{dt}\geq-0.7z+\frac{4x}{1+0.1x}z. \end{aligned} $$

Through a simple calculation, we have $E^{\ast}= ( {1.2454,0.1523,0.9467} )$. Firstly, we get ${\tau_{0} =2.31}$ when $\tau_{1} =\tau_{2} =\tau\ne0$. Then we have $\tau_{10} =2.58$ when $\tau_{2} =0$. Next we obtain ${\tau_{20} =2.945}$ when $\tau_{1} =0$. Finally, by regarding $\tau_{2} $ as a parameter and letting $\tau_{1} =2.5$ in its stable interval, we prove that $E^{\ast}$ is locally asymptotically stable for $\tau_{2} \in ( {0,\tau_{30} } )$ and unstable for $\tau_{2} >\tau_{30} $.
